# A Case of Bilateral Hearing Loss

**DOI:** 10.5811/cpcem.2020.9.48949

**Published:** 2020-10-20

**Authors:** Boris Ryabtsev, Matthew Slane

**Affiliations:** Kendall Regional Medical Center, Department of Emergency Medicine, Miami, Florida

**Keywords:** Bilateral hearing loss, posterior stroke, vertebral artery occlusion

## Abstract

**Case Presentation:**

A 53-year-old male presented to the emergency department with acute onset of bilateral hearing loss as well as vertigo and severe vomiting. The Head Impulse– Nystagmus–Test of Skew exam was indicative of a central neurologic process. Computed tomography angiogram of the head and neck revealed near-total bilateral vertebral artery occlusions in the second and third segments. The patient was admitted for further evaluation; subsequent magnetic resonance imaging revealed multiple areas of infarction in the cerebellar hemispheres, medulla, and occipital lobes.

**Discussion:**

This case describes a unique presentation of a posterior stroke. Common symptoms include vertigo, loss of balance, and vomiting. However, bilateral hearing loss as a prominent symptom is uncommon. Imaging revealed a rare finding of bilateral vertebral artery occlusion.

## CASE PRESENTATION

A 53-year-old male presented to the emergency department by ambulance complaining of acute onset of severe vertigo, vomiting, and bilateral hearing loss. Symptoms began six hours prior, and progressively worsened. The patient was unable to walk due to severe ataxia. He denied any significant past medical or surgical history but reported smoking and a family history of stroke. No recent trauma or injury was identified. During evaluation, patient was vomiting and extremely hard of hearing. He was hypertensive at 147/78 millimeters of mercury, all other vital signs were within normal limits. The Head Impulse–Nystagmus–Test of Skew (HINTs) exam was positive for bidirectional horizontal nystagmus and test of skew bilaterally. The patient demonstrated abnormal finger-to-nose and heel-to-shin testing bilaterally. Non-contrast computed tomography (CT) of the brain showed no acute intracranial hemorrhage; however, CT angiograms of the head and neck demonstrated abnormalities that confirmed the diagnosis.

## DISCUSSION

**Image 1:**
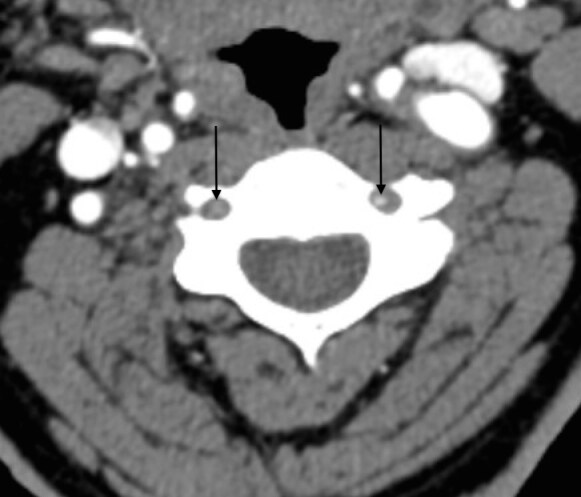
Axial computed tomography image showing decreased vertebral artery flow bilaterally at the level of the third cervical vertebra.

**Image 2:**
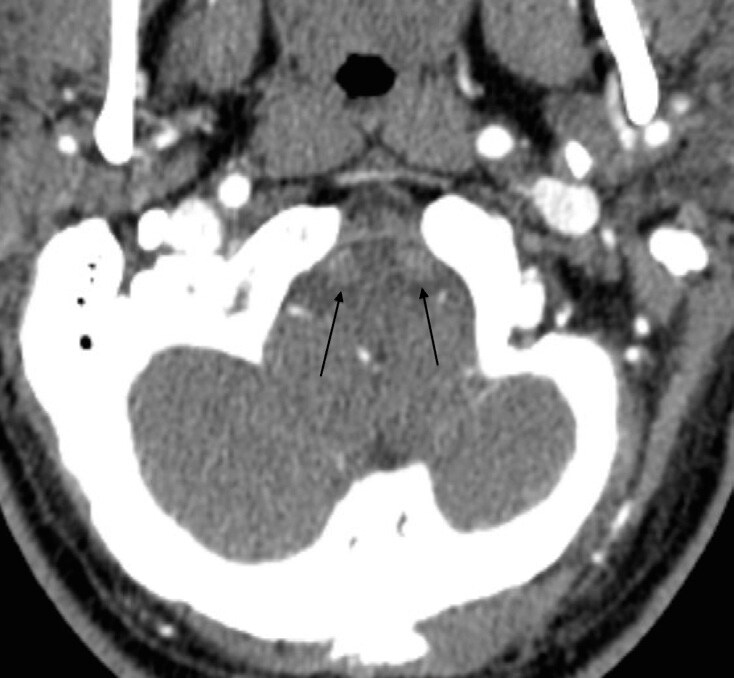
Axial computed tomography image showing decreased vertebral artery flow bilaterally at the level of the cerebellum.

**Image 3:**
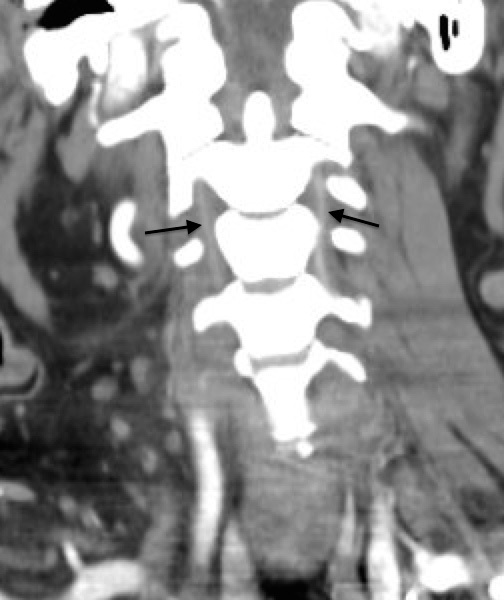
Coronal computed tomography image showing decreased vertebral artery flow bilaterally.

Computed tomography angiograms demonstrated near-total bilateral vertebral artery occlusions in the second and third segments (Images 1–3) without dissection.

Subsequent magnetic resonance imaging revealed multiple acute infarctions of bilateral cerebellar hemispheres, right aspect of medulla, and bilateral occipital lobes. The patient was started on aspirin and clopidogrel per neurology. Cardiology evaluation did not reveal a cardioembolic source. The patient was additionally diagnosed with hypertension and diabetes, which were likely involved in the disease process.

Posterior circulation strokes make up 10–20% of all ischemic events in the brain.^1^ Common presentations include vertigo, loss of balance, vision loss, slurred speech, nausea, and vomiting. Hearing loss may occur due to decreased blood flow through the internal auditory artery, a branch of the anterior inferior cerebellar artery, which supplies the cochlea. However, bilateral hearing loss as a prominent symptom is rare. Studies show the incidence of bilateral hearing loss in vertebrobasilar disease to be less than 2%.^2^–^4^ The HINTs exam was crucial to differentiate central vs peripheral etiology of the patient’s vertigo.^5^

CPC-EM Capsule

What do we already know about this clinical entity?

*Posterior strokes make up a considerable number of all ischemic events in the brain. Common symptoms include vertigo, vision loss, slurred speech and nausea*.

What is the major impact of the image(s)?

*The images show near-total bilateral vertebral artery occlusions, an extremely rare presentation of a posterior stroke*.

How might this improve emergency medicine practice?

*This case of a patient presenting with bilateral hearing loss may help the physician identify a posterior stroke based on a patient’s hearing changes*.
